# DNA barcoding in diverse educational settings: five case studies

**DOI:** 10.1098/rstb.2015.0340

**Published:** 2016-09-05

**Authors:** Heather J. Henter, Ralph Imondi, Karen James, Diana Spencer, Dirk Steinke

**Affiliations:** 1 Natural Reserve System, University of California San Diego, La Jolla, CA 92093, USA; 2 Coastal Marine Biolabs, Ventura, CA 93001, USA; 3 MDI Biological Laboratory, Bar Harbor, ME 04609, USA; 4 Tulsa Community College, Tulsa, OK 74133, USA; 5 Centre for Biodiversity Genomics, Biodiversity Institute of Ontario, University of Guelph, Guelph, Ontario, Canada N1G 2W1

**Keywords:** DNA barcoding, biodiversity, education, Next Generation Science Standards, citizen science, community college

## Abstract

Despite 250 years of modern taxonomy, there remains a large biodiversity knowledge gap. Most species remain unknown to science. DNA barcoding can help address this gap and has been used in a variety of educational contexts to incorporate original research into school curricula and informal education programmes. A growing body of evidence suggests that actively conducting research increases student engagement and retention in science. We describe case studies in five different educational settings in Canada and the USA: a programme for primary and secondary school students (ages 5–18), a year-long professional development programme for secondary school teachers, projects embedding this research into courses in a post-secondary 2-year institution and a degree-granting university, and a citizen science project. We argue that these projects are successful because the scientific content is authentic and compelling, DNA barcoding is conceptually and technically straightforward, the workflow is adaptable to a variety of situations, and online tools exist that allow participants to contribute high-quality data to the international research effort. Evidence of success includes the broad adoption of these programmes and assessment results demonstrating that participants are gaining both knowledge and confidence. There are exciting opportunities for coordination among educational projects in the future.

This article is part of the themed issue ‘From DNA barcodes to biomes’.

## 1. Introduction

Many calls have gone out to provide more opportunities to participate in scientific research for both students and the general public. For example, the Next Generation Science Standards (NGSS) in the USA recommend that all primary and secondary school students (ages 5–18) understand science as it is practised in the real world [1]. The landmark *Vision and change in undergraduate biology education* report [2] emphasizes how transformative research experiences can be for post-secondary students. Citizen science groups advocate participation of the general public in science to both produce invaluable data and to increase informed civic involvement in science policy [3]. In sum, these recommendations suggest that research participation in both formal and informal settings could increase science interest, literacy and engagement.

These ideas are soundly based in educational theory. When individuals participate in an original research project, they are engaging in an authentic task situated in a social context, consistent with situated learning theory [4]. Participants interact within a community of practice, a group of people working towards a common goal, in this case a research question. Professional scientists, teachers, laboratory members, peers or volunteers encourage reflection, and participants gain insight into the values, practices and cultural norms of the scientific community. This differentiates research participation from a simple ‘hands-on’ experience. The prediction from theory is that research participants will gain a sense of belonging to the scientific community, an appreciation of scientific values and a firmer science identity [5,6]. Lave & Wenger [4] emphasize ‘legitimate peripheral participation’ in which novices initially work on the periphery and move towards more full participation as they gain skills and experience.

### (a) DNA barcoding

DNA barcoding to document biodiversity has provided research opportunities across a range of educational contexts and scales. In DNA barcoding, a short, standardized region of the genome is used to differentiate species [7]. In animals, a 650 base-pair region of the mitochondrial cytochrome c oxidase 1 (*CO1*) gene is used. This region is variable enough to distinguish species in most cases, yet short enough to be sequenced cheaply. An international effort to ‘barcode’, or create a reference library of barcodes from known species, is ongoing, with 500 000 species already in the Barcode of Life Database (BOLD, http://www.boldsystems.org, [8]). Various taxa or geographical areas have received extra attention, such as the effort to barcode the world's butterflies and moths (Lepidoptera), with over 100 000 species barcoded to date, two-thirds of the 150 000 described species. Barcoding of other taxa is just beginning. We posit that using DNA barcoding to document biodiversity is a natural research topic for educational settings for three reasons.

First, the science is compelling. One of the most underappreciated aspects of biodiversity is that, despite 250 years of taxonomy, most eukaryote species remain undocumented by science. Current estimates of the total number of species range from 5 to 10 million, but fewer than 2 million species have been named or described [9,10]. This knowledge gap is not evenly distributed among taxa. Although most vertebrate animals are known, Hamilton *et al*. [11] suggest that 70% of arthropod species await description. Given current rates of taxonomic classification, Mora *et al*. [10] estimated that it would take over 1000 years to describe the remaining eukaryotic species. With accelerated extinction rates [12], many species will be extinct long before we know they exist [10]. Using DNA barcoding students and citizen scientists alike can help address the biodiversity knowledge gap. Online tools are currently available that allow non-professionals to contribute high-quality data to the international effort.

Second, the concept and practice of DNA barcoding are relatively straightforward compared to other disciplines and techniques in genetics, genomics and molecular biology [13]. That DNA can be used to discover the provenance or identity of a biological object is generally well known, thanks in part to the popularity of forensic science on television. Moreover, the steps of the DNA barcoding workflow—collection and identification of specimens, DNA extraction, PCR, DNA sequencing and contribution or comparison to a database—are also relatively straightforward compared to other laboratory workflows in biology.

Third, this workflow naturally divides into independent modules. Practitioners of DNA barcoding in education can focus on one module or the whole process of DNA barcoding. For example, as described below, younger students can focus on collecting specimens but send those specimens elsewhere to generate DNA data. Other groups do not collect specimens but focus on laboratory work with specimens collected by collaborating natural resource managers. Most post-secondary participants collect their own specimens and do most of the laboratory work, but send PCR products to a commercial facility for DNA sequencing. And finally, it is possible to just work with data that is already in BOLD. DNA barcoding research can be adapted to fit a variety of educational contexts.

Below we describe how DNA barcoding has been adopted by primary schools (students age 5–12), secondary schools (students age 12–18), a community college (a post-secondary 2-year school), a university (a post-secondary school offering baccalaureate degrees) and an informal science education programme for the general public. Each setting has carried out the research in different ways, for different purposes, and has reported different participant outcomes. But each project has contributed new data to the international effort to inventory the world's biota.

## 2. DNA barcoding in education

### (a) School Malaise Trap Project: grades 4–12

The American Association for the Advancement of Science encourages primary and secondary school teachers to offer their students the ability to explore nature from a scientist's perspective and promotes students' active participation in inquiry-based learning. The goal is that students understand and feel a part of the development of knowledge [14]. Motivated by the perception that these teachers experience difficulty in keeping up to date with science innovation and discovery, some universities have launched outreach programmes aimed at improving science education for the participating students and leading change in the teaching of science curriculum (e.g. [15,16]).

The School Malaise Trap Program, run by the Centre for Biodiversity Genomics (CBG), is an example. Primary and secondary school students spend a large portion of their lives at school, and this includes interacting with the school's natural environment. Students aged 7–14 will complete approximately 8000 h of instruction within public academic institutions [17]. The School Malaise Trap Program is meant to encourage students in grades 4–12 (age 9–18) to actively explore, question and evaluate this world in which they spend so much time—starting with their schoolyard. Across Canada, students and educators use Malaise traps, tent-like structures that capture flying insects [18], along with DNA barcoding to explore the insect diversity around their schools. The project was initiated in 2013 and to date over 250 schools and almost 15 000 students have participated. During the programme, students are introduced to multiple environmental, science, technology, engineering and math (E-STEM) disciplines while experiencing field biology and exploring DNA barcoding through participatory and inquiry-based scientific research.

To begin the programme, each school receives scientific research material including the Malaise trap and sampling bottles to collect insects for a specified two-week period. To build critical thinking skills, students collect, organize and record daily temperature and weather data, as well as surrounding habitat data and sample bottle catch volume. Students are then asked to formulate questions, hypotheses and predictions regarding the relationships between the catch volume and these other variables. Further investigations to answer these questions reinforce the students' ability to research, analyse and justify their conclusions while using a variety of skills (linguistic, numeric, symbolic, media) to communicate ideas and results.

Once sampling is complete, the schools return the specimens to CBG to be identified with DNA barcoding. All the specimen data are uploaded to BOLD. To conclude the programme, individual trap results are emailed to the teacher; these results include a personalized school report containing a list of the species caught at their school and an image library of those species. A detailed final programme report is also included that provides an overview of the data obtained across all the participating schools and traps, including site comparison information and interesting specimen discoveries. Analysing these reports enables the students to answer their previously formulated hypotheses as well as predict potential impacts of their findings on society.

While managing the Malaise trap during the specified two-week period, students are encouraged to work collaboratively and to take ownership and responsibility of trap deployment and monitoring. Educators note that the students have a sense of ownership of the project that they share with their school and community, ultimately fostering accountability, which helps to ensure success of their research project. Furthermore, in surveys conducted at the end of each project 91% of educators stated that their students demonstrated a positive response to the programme, contributing to the programme's success and expansion.

CBG provides comprehensive lesson plans to each school. These plans address specific expectations across elementary and secondary curricula as well as supplementary cross-curricular activities and lesson extensions. The programme is designed to emphasize critical thinking skills across several Canadian core curriculum subjects including biological and environmental science, mathematics, English, technological studies and geography. Additionally, this programme immerses students in the subjects of environmental literacy, global awareness and citizenship via the enhancement of environmental stewardship and leadership skills. Through this participatory approach to scientific learning, the School Malaise Trap Program provides students and educators with a real sense of discovery and collaboration by contributing valuable data to BOLD. To date, the programme has sequenced over 68 000 insect specimens representing over 6500 individual species. Additionally, over 1000 of the individual species collected so far have been new to BOLD.

### (b) Barcoding Life's Matrix: professional development for secondary school teachers

New science education standards for students age 5–18 have recently been adopted by a number of US states. The NGSS promote a deeper understanding of science content and its application, and appreciation for the interconnected nature of science as it is practised and experienced in the real world [1]. These broader aims form the pedagogical cornerstones of the Barcoding Life's Matrix program, an interdisciplinary research education programme launched in 2011 by Coastal Marine Biolabs (CMB) [19]. Now national in scope, the project establishes a model for NGSS implementation by engaging 11th- and 12th-grade secondary school students (age 16–18) and their teachers in building the global DNA barcode reference library. Student learning is organized around the assembly, analysis, validation and publication of reference DNA barcode records for commercially and/or ecologically important target specimens. Both quantitative and qualitative measures are used to examine the impact of the experience on the knowledge and attitudes of high school students and teachers. Although out-of-school residential research experiences for students constitute an important component of the programme, we limit our discussion below to the project's professional development component.

Professional development unfolds in a 12-month learning cycle that begins with a 6–8 day residential summer institute at CMB. During each institute, scientists model the delivery of content and engage teachers in the work that students will ultimately conduct during the three-week student classroom experience at their high schools. Teachers and students carry out all aspects of the DNA barcoding workflow, including the assembly, validation, publication and analysis of reference barcode records from data that they generate at the bench. To support student learning, the project uses multimedia instructional resources that were developed collaboratively by scientists and a small team of early adopters from a local school district. These resources form 16 discrete instructional units that combine related NGSS performance expectations along a wide spectrum of advanced life science themes and topics ranging from biological diversity to molecular life science to next-generation sequencing technology and metabarcoding. When the teachers implement the project the following school year, these materials are deployed through a content management system that resides on the Education and Barcode of Life Community Web Portal (eBOL), an open-access website aimed at advancing DNA barcoding as a new tool to enhance life science teaching and learning (www.educationandbarcoding.org). The system includes tools for student assessment and provides important real-time feedback on teacher and student engagement in project-related instructional resources. The system also provides a streamlined mechanism for science professionals and educators to contribute various forms of supplemental digital assets (e.g. scientific illustrations and images, video animations, etc.) to aid classroom instruction.

The opportunity for students to apply twenty-first century science practices and process skills associated with ‘big data’ science (e.g. data discovery, mining, annotation, attribution, validation, visualization, analysis, sharing and publication) is regarded by teachers as a unique strength of the programme. These activities are supported by the BOLD Student Data Portal (BOLD-SDP: http://www.boldsystems.org/index.php/SDP_Home: [19]). BOLD-SDP allows novice researchers to contribute data to the international research effort while maintaining data quality and meeting the needs of the classroom instructor. For example, instructors can easily track student online activity and generate reports on task completion. The process of barcode record assembly is simplified into four distinct steps (upload specimen and collection data, upload images, upload trace files and edit/add sequence) so that different participants can collaborate on the creation of a barcode record during one or more standard class periods. To safeguard the fidelity of student-generated barcode data BOLD-SDP employs a hierarchical, three-tier data validation protocol through which students and teachers, project staff and professional data managers share in the responsibility of evaluating student barcode records for compliance with data standards [19]. Through BOLD-SDP, validated barcode records appear in BOLD with full student attribution. Finally, BOLD-SDP includes data visualization and analysis tools that allow students to apply a spectrum of cyber-enabled science practices and process skills as they organize, validate and explore their barcode data during the classroom experience.

At present, secondary school teachers and students have assembled and contributed over 1600 reference barcode records to BOLD. These include 113 marine species of bony and cartilaginous fishes, molluscs, echinoderms and cnidarians. Many of these records are associated with ongoing marine biodiversity inventories conducted by the Channel Islands National Park, the Cabrillo Aquarium, Moss Landing Marine Laboratory and the Alaska Fisheries Science Center. Tissue samples from taxonomically verified vouchers were processed and maintained in the CMB lab, and subsequently distributed to classrooms along with pre-assembled barcoding kits containing the necessary reagents and consumables. Students extract and amplify DNA from their samples and send PCR products to a commercial facility for sequencing. Image files and corresponding metadata for each specimen (20 per class), as well as trace files generated from student-generated *CO1* amplicons, were organized into ZIP archives and sent to teachers electronically approximately one week prior to the assembly of barcode records in BOLD-SDP. Student barcoding efforts have recently expanded to include insect specimens obtained in connection with a large-scale restoration effort on Santa Rosa Island, the second largest of California's northern Channel Islands.

Over the past 3 years, 61 secondary school science teachers from 23 states spanning both US coasts (including Alaska and Hawaii) have participated. Teachers implemented the three-week classroom experience as a research component in a range of general and elective courses (e.g. standard, honours, and advanced placement biology, marine biology, chemistry, and specialized biotechnology and research courses), resulting in the engagement of over 2700 16–18 year-old students in the programme.

Survey data suggest that both teachers and students gained science content and process knowledge and expressed more positive attitudes about science after participating in the programme. Many of these gains persisted over time [20]. Additionally, qualitative interview data revealed that the project changed teachers' perceptions of what their students are capable of learning and achieving, and their perspectives on the depth of knowledge required to effectively teach science (an extremely important outcome given the emphasis of the NGSS on depth and academic rigour). Teachers noted several aspects of the project that they felt were important for classroom teaching and learning: (i) direct interactions with scientists during the week-long professional development institutes, (ii) strict coherency between professional development and classroom instruction, (iii) real-time support before and during enactment of the classroom experience, and (iv) the opportunity for students to contribute data to an authentic scientific initiative, which was uniformly regarded as central to student interest, excitement and ownership of their learning. Teachers also reported that the programme engaged their students more than usual, stimulated the interest of students who do not typically find science interesting, and heightened student motivation to explore STEM careers or undergraduate studies. Based on these and other positive outcomes, plans are underway to scale the project across a greater range of educational contexts and settings and to develop additional resources to support this expansion [20].

### (c) The San Diego Biodiversity Project: a university example

Probably the most familiar model of non-professionals engaging in research is the tradition of university students working in a professor's research laboratory. These research apprenticeships are standard at universities, but not all science students can or will participate [21,22]. To make research experiences more inclusive, a new model of course-based research is gaining acceptance [23]. There is evidence that both apprenticeship and classroom research produce equivalent outcomes [24]. In general, research experiences can increase students' content and conceptual knowledge, self-perception of technical and analytical skills, understanding of the nature of science, sense of project ownership, scientific confidence, career clarification, science identity, and persistence or intent to persist in science [6,25].

At the University of California, San Diego undergraduate students in biology courses use DNA barcoding to generate novel biodiversity data that they communicate to the scientific community through BOLD. The goal is to create an inventory of invertebrate biodiversity at the Scripps Coastal Reserve, one of the properties in the network of wild lands managed by the UC Natural Reserve System. The focus is on invertebrate animals because they are so poorly known [11]. The classes often test specific hypotheses as part of the overall goal of documenting invertebrate biodiversity. Enthusiastic undergraduates or postgraduate MSc students have followed through on several questions that were generated in these courses. For example, students in an undergraduate course determined that DNA barcode variation could differentiate honeybee mitochondria from Africa versus Europe. An MSc student confirmed this result with additional molecular markers and then showed that BOLD can be used as a tool to follow the invasion of the Africanized honeybee across the globe [26]. Similarly, students in an undergraduate course used DNA barcoding to determine the identity of polychaete species in the sandy beach; an MSc student continued this work to examine the effect of beach management on species diversity [27].

The classroom component of the UC San Diego project is a two-week module embedded in two upper division laboratory courses with an average total enrollment of over 500 students per year. Students in an ecology field course collect specimens, record the ecological data, take high-resolution photographs, document vouchers and then pass specimens to the students in a molecular biology class. Those students extract and amplify the DNA and send the PCR product to a commercial facility for sequencing. The DNA sequence data come back to the students in the last weeks of the term and the molecular biology students create a consensus sequence for the forward and reverse reads. Students in both courses have a chance to work with the final data. They compare their sequences to the existing records in BOLD to identify species or determine whether they are new to the database. They build DNA-based phylogenetic trees depicting the evolutionary relationships among their specimens, and they compare intraspecific and interspecific variability [28].

Similar to the secondary school students in the Barcoding Life's Matrix program, university students contribute data to BOLD through a student data portal, but starting in summer 2015 students use the University Student Data Portal (BOLD-UNI SDP, http://uni.boldsystems.org/index.php/SDP_Home). The BOLD-UNI SDP differs from the BOLD-SDP in that it focuses on the needs of the post-secondary classroom. Students are expected to enter additional data, such as voucher status and location, habitat data and species associations, and the university portal allows data to be shared between classes and institutions. Similar to its BOLD-SDP counterpart, there are several tools embedded in the BOLD-UNI SDP that enable students to address ecological and evolutionary questions with the data, such as tree-building, species accumulation curves or barcode gap analyses. To ensure the integrity of the data, data validation on the BOLD-UNI SDP is identical to that on BOLD-SDP described above. Validated data appear on BOLD with full student attribution.

From September 2012 through June 2015, over 1700 undergraduate students at UC San Diego contributed original data to BOLD-SDP. During this time period, students successfully produced DNA sequence data for 591 of 839 specimens (70%). A consensus sequence was created for 490 specimens, 58% of the total. As an example, in the winter term of 2015, 15% of the specimens that were successfully sequenced were species new to the BOLD database. There are now 221 UC San Diego student-generated individual records, representing 53 species, publically available on BOLD. That number will increase as the specimens work their way through the validation process. Records can be found by searching the Public Data Portal of BOLD using the search term ‘UCSD’.

Surveys and focus groups have shown, similar to the other examples described above and below, that students' attitudes changed after taking courses that included original DNA barcoding research. At the end of the term, undergraduate students were more likely to think of themselves as scientists [29]. The most striking result of the focus groups was how proud students were that they had made a contribution to science. Additionally, the survey data showed that there was a disproportionately positive effect on female students compared to male students [29]. Previous work suggests that female students place more importance on making a contribution or addressing local problems [30–34], which could help explain these results.

### (d) Tulsa Community College: a post-secondary 2-year school

In the USA, nearly half of all post-secondary students are in community colleges [35]. These 2-year schools offer programmes that are either vocational or preparation for university and serve as a gateway to education beyond what is compulsory for many low income, minority and first-generation students. Community colleges have a diverse student population with more than 50% black and 60% native American students. The average student age is 29 years old with approximately 80% of the students balancing part-time to full-time courses with part-time to full-time employment [36].

Community colleges play a pivotal role in STEM education, given that approximately 50% of all science, engineering and health baccalaureate degree recipients attend community college at some point in their studies [37]. However, economic projections determine that an additional one million STEM professionals, above current levels of training, are needed over the next decade for the US to remain competitive in science and technology [38].

Although much attention is given to attracting students to STEM, retaining students is also a challenge. Overall, less than 40% of students entering post-secondary programmes with a STEM major complete a baccalaureate degree, and less than 25% of underrepresented minority students do so. Increasing overall retention to 50% would generate three-quarters of the targeted one million STEM graduates needed [38]. Again, community colleges can play an important role. Most students who drop out of science do so in the first 2 years of post-secondary training [39]; thus, it is not surprising that research experiences have the highest impact on student retention early in their post-secondary experience [40].

Tulsa Community College (TCC) is Oklahoma's largest community college, with 27 000 students. Approximately 35% of the student population belongs to an underrepresented minority. Similar to the 4-year university example above, research is integrated into the curriculum. The TCC Biotechnology Program offers an Associates of Science degree in Biotechnology for transfer to baccalaureate-awarding institutions and a Certificate in Biotechnology, a workforce development program used primarily by post-baccalaureate students. The programme includes a series of research experiences available to the students, with stand-alone courses, apprenticeships, internships and course-embedded investigations.

A three-week module in DNA barcoding is embedded in the molecular biology course taken during the student's last semester on campus. DNA barcoding was chosen because it can be adapted to different organisms while teaching the required laboratory and bioinformatics skills. Projects have focused on arachnids, biting midges, angiosperms, aquatic metazoans, scat collected at the zoo, and ancient oak forest habitats endemic to the area. Students are involved in the collection and identification of the organisms in collaboration with the scientific community, including conservation specialists, veterinarians, zoologists and botanists who document specimen identity. This experience with professionals demonstrates the interconnectedness of the science community, the specificity of knowledge and the value of collaboration. Students' family members were also involved; for example, they sent specimens of ants with descriptions of habitat to compare to the local ant fauna. This citizen science model increased communication skills and excitement as students discussed their project around the dinner table.

As part of the module, students share a peer-reviewed paper with the class that explains previous barcoding work related to a specific organism of interest. Similar to the 4-year example, the laboratory skills taught include field collection, DNA extraction and amplification, and preparation of their sample for the external sequencing facility. With assistance from staff from the Oklahoma Idea Network for Biomedical Research Excellence, students edit and analyse the DNA sequence data. For example, TCC students use MEGA 6 [41] for phylogenetic tree reconstruction, and they visualize the protein structure of the gene using Cn3D [42]. As a result, students gain expertise and competency to investigate any gene region.

The students often express an interest in a career in bioinformatics. As one student explained, ‘…Bioinformatics presented itself as a definite and new area of interest for me that I will continue to feed my curiosity and allow me to make specific progress toward my educational career’. Students and professor often become colleagues while analysing and conjecturing about confounding results. Why was rat DNA found in the Komodo dragon scat? Why was the dragon fly identified as midge? Why was the fish purchased at the store counter labelled incorrectly? Students frequently take the lead in interpreting their own data.

The final piece of the module includes a poster presentation to the community in a science forum. Interactions with students suggest that this experience greatly increases their confidence in science and in themselves. Our interpretation is that the opportunity to explain knowledge that they themselves have produced gives students greater science identity and ownership of the data. Statements from students make it clear that they feel a part of the community of scientists.

DNA barcoding is an excellent fit for the community college classroom because students can learn important skills and address interesting research questions. Accordingly, TCC is expanding the use of DNA barcoding in the curriculum. A related computer laboratory investigation is offered in the majors biology course and a national database of barcoding investigations through community colleges is being developed through the Community College Undergraduate Research Initiative (http://www.ccuri.org/). There is also a consortium of 2- and 4-year schools in southern California that collaborate on incorporating DNA barcoding into the curriculum.

### (e) Biotrails: Informal Science Education

Students and members of the public can learn science in informal settings, including museums, parks, homes and virtual environments [43,44]. Public participation in scientific research, or citizen science, is one such opportunity [45]. Citizen scientists routinely participate in projects on climate change, invasive species research, ecological restoration, conservation and many types of environmental monitoring [46]. Notable citizen science projects within ecological and environmental science include Audubon's Christmas Bird Count (birds.audubon.org/christmas-bird-count), eBird (ebird.org), Project BudBurst (budburst.org), Nature's Notebook (usanpn.org), and Notes from Nature (notesfromnature.org). Not only have these projects produced valuable data [47], but they also have reported personal and community-wide benefits for participants. Common participant outcomes include increased knowledge, heightened environmental awareness and changes in attitudes and behaviours, such as increased political participation [48]. For example, in several cases citizens influenced resource management decisions, empowered by their own data [48–51]. Consistent with theory [5,6], these actions imply an increased sense of affiliation to the scientific community.

In addition (and integral) to its educational benefits, citizen science can expand the scientific workforce to generate data to meet difficult, large-scale or computationally intensive scientific challenges [45,52]. One of these challenges is understanding and predicting how organisms respond to climate change and other human impacts on the environment [53,54], which requires ‘a new kind of ecology…predicated on scaling up efforts, data sharing and collaboration’ [55, p. 5].

In ecology and environmental science, however, data collection very often requires the identification of biological specimens, a difficult and labour-intensive task compounded by a dearth of taxonomic experts. This taxonomic impediment limits the capacity of both research and its applications [56]. The taxonomic impediment is compounded in the context of citizen science. Projects that engage the public in the collection of specimens, including bioblitzes [57], rely heavily on professional taxonomists to identify specimens, so the taxonomic impediment remains in place. This reliance can preclude the involvement of citizen scientists in subsequent steps of scientific investigations like analysis and interpretation, and can reinforce a potentially damaging ‘us vs. them’ attitude, in which citizen scientists are not in possession of the knowledge required to participate in science [58].

BioTrails (mdibl.org/education/biotrails/), a program of the Mount Desert Island (MDI) Biological Laboratory, Acadia National Park, and the Schoodic Institute, overcomes the taxonomic impediment through DNA barcoding to scale up environmental citizen science. BioTrails participants observe and collect specimens, identify and/or sort them to phylum (animals) or divisions (plants), then submit them for identification through DNA barcoding at the MDI Biological Laboratory. DNA-validated identifications are then delivered to a range of collaborators including environmental researchers, conservationists and managers, with the particular environmental data needs that prompted the collections in the first place.

As part of this pilot project, the representation of the Acadia region plant and animal species in the DNA barcode reference library within BOLD has also been improved, to serve as an identification resource for citizen-scientist-collected specimens. The reference library for the plants of Acadia National Park is particularly close to completion, with fewer than 10 out of nearly 900 species remaining. The marine invertebrates of the Acadia region is another area of focus, with approximately three-quarters of the approximately 800 species now with at least one reference sequence in BOLD.

BioTrails has been implemented on a small scale so far, with approximately 50 adult volunteers participating as citizen scientists in an initial pilot project from 2013–2015. This project produced preliminary learning research that will be published elsewhere, but addresses the specific challenges of short-duration, technology-assisted engagements such as those that BioTrails offers. In the coming years, the project team, recently expanded to include the Maine Mathematics and Science Alliance, the University of Maine and the Bigelow Laboratory for Ocean Sciences, plans to engage over 1000 participants in the Acadia National Park region of coastal Maine, ultimately spreading to other regions of the USA to reach many more participants.

BioTrails will soon be implemented via *BioTrails Basecamp*, a user-friendly online platform and mobile app that will enable and support large-scale data generation and research in both informal environmental science learning and environmental science. Powered by Anecdata (anecdata.org), a next-generation online citizen science tool, *BioTrails Basecamp* will be for everyone: informal STEM learning researchers and practitioners, environmental scientists, project participants and others will be able to generate and/or analyse large-scale environmental and learning data. It will include a species identification decision support tool to integrate multiple technologies for species identification: not only DNA barcoding, but also online tools and apps such as the iNaturalist (inaturalist.com).

## 3. Discussion

DNA barcoding has been used successfully in a variety of educational contexts, both those described here and elsewhere [59–61]. The power of DNA barcoding in education was acknowledged in 2013 when this approach won the Prize for Inquiry-Based Science Instruction [62]. In the projects we describe, there are a number of commonalities that illustrate why this is an effective educational strategy.

First, participants are engaging in scientifically valid and important tasks situated in a social context [4]. They become part of a community of practitioners. In the School Malaise Trap Program, students in grades 4–12 interact with scientists at CBG. Secondary school students involved in Barcoding Life's Matrix barcode samples collected by natural resource managers involved in marine conservation. University students at UC San Diego interact with UC Natural Reserve managers on the common goal of creating an invertebrate inventory for a nature reserve adjacent to campus. Post-secondary students at TCC work with local professionals, from botanists to veterinarians, to identify the specimens they collect. BioTrails participants collect specimens to meet a range of environmental data needs. And when participants in any of these programmes upload data to BOLD, they contribute to an international scientific project. Although contributing data to a scientific database is not the equivalent of publishing data in a peer-reviewed scientific paper, uploading data to BOLD is an inclusive process in which each individual can be given credit for the tasks they accomplish. Thus, learners participate in the culture of generating new scientific knowledge and they become peripheral members of the broader scientific community. All the programmes report, qualitatively or quantitatively, an increase in pride, confidence and science identity in their participants, and we attribute these outcomes to student participation in the broader scientific effort. A UC San Diego undergraduate student summed it up by saying, ‘…having that site (*BOLD*) published and out there for other people to see. That's kind of a cool thing and made me definitely think that I am a scientist’ [29].

Another commonality is that most of the programmes use DNA barcoding to address content and skills required of their students. The School Malaise Trap Program addresses core content for Canadian primary and secondary school students, the Barcoding Life's Matrix program is aligned with the NGSS in the USA, and TCC uses DNA barcoding to teach basic laboratory and bioinformatics skills needed for the biotechnology certification. DNA barcoding provides a solid basis for science instruction and addresses current educational standards because it bridges disciplines, as exemplified by the collaborations between ecology and molecular biology classes at UC San Diego, the math and science skills required to test predictions about catch volume in the School Malaise Trap Program, and the interdisciplinary life science curriculum unit advanced by the Barcoding Life's Matrix program. Thus, it has the potential to foster integration between what students perceive as disparate courses [61].

Although the projects described here share these fundamental similarities, they differ in their emphasis. This flexibility is owing to the modular nature of the DNA barcoding workflow, which is made up of interrelated field, laboratory and informatics components. Participants can focus on specimen collection and documentation, DNA extraction and amplification, bioinformatics, data analysis or a combination of these activities. The workflow compartmentalization is also what enables collaboration among different groups, whether that is within interdisciplinary classes or across multiple institutions or agencies.

The authenticity of barcoding-based research experiences introduces several challenges for the classroom. In most of our examples, the teaching staff manage high teaching loads or large numbers of students, which precludes the considerable investment of time required for preparation and implementation of a research project. For example, contributing data to the scientific community is a critical component of the DNA barcoding enterprise, but quality control (i.e. validating specimen data before they appear in the BOLD public data portal) is a time-consuming process. Nevertheless, we expect that as more educators get involved in DNA barcoding and as our collaborative network expands accordingly, more creative solutions to help address time constraints and other barriers will emerge. In two of the examples described here, Barcoding Life's Matrix and the School Malaise Project, the project leaders received extramural financial support to help primary and secondary school teachers minimize the time demands outside of the classroom.

A second challenge is the technical difficulty experienced by educators who lack laboratory expertise or familiarity with the diverse taxa encountered by students in biodiversity research. Although general procedural instructions are widely available, detailed technical information for specific taxa (e.g. tissue selection, primer selection, cycling conditions, etc.) is only found in the scientific literature, which is prohibitively time consuming to explore. A collaborative strategy involving ‘crowdsourcing’ summaries of these technical details, specific to educational settings, could be a useful tool to circumvent this challenge. For example, an open-access wiki with protocols and a summary of the best primers to use for common orders of invertebrate animals would be invaluable for instructors.

A third challenge is the cost. At UC San Diego, we spend $15 per specimen, which includes $5 for disposable laboratory supplies and $10 for bidirectional sequencing at a commercial facility. These funds come out of student laboratory fees. This is feasible for some post-secondary schools, but not all. Costs for participants of the School Malaise Trap Program averaged $1000 per school, which were entirely covered by third-party funding. All of the projects described here have been supported indirectly or directly by grant funding, which must be sought again every few years. A sustainable source of funds is clearly needed.

Despite these challenges, DNA barcoding presents rare opportunities for collaboration. Because DNA barcoding has been so widely adopted by educational institutions with large numbers of students, the potential exists to collaborate on a coordinated, rigorous and targeted evaluation of student outcomes. These outcomes might include content knowledge and science process skills, attitudes such as science identity and self-efficacy, retention of students in STEM and engagement of the public in science. There is a rapidly growing literature assessing the impact of an original research experience in general (reviewed in [23,25]), but there are few data specific to DNA barcoding in different educational settings [19,29,61]. The value of assessments could be maximized if varied institutions work together to develop and validate evaluation instruments that could be used to generalize across contexts. Such an effort will be a key to document and inform future success. The importance of evidence-based approaches in education is increasing, especially when federal funds are involved [63].

Another exciting and challenging opportunity for collaboration involves the engagement of multiple student and participant groups on a joint research project that would benefit by data gathered across multiple scales. This could be a competition to submit a certain number of species in a year, or it could be a hypothesis-driven research question. This is a long-term goal that could only be realized with an integrated community of instructors and practitioners. That integration could happen in a number of ways. The current eBOL website was designed to serve as an umbrella teaching commons for multimedia curriculum resources and student assessment tools, a source of technical support specific to educational projects and a registry for project descriptions and data summaries. The BOLD-UNI SDP allows groups at any institution to share data, but this opportunity has yet to be used. A common goal could motivate students to share data. A list of instructors and practitioners at all levels that are using DNA barcoding in their formal or informal classrooms and projects would facilitate collaboration. Finally, the international barcoding community currently hosts a biennial conference for researchers that includes an education component; increasing the number of educators at this conference, or developing an independent education-oriented meeting or workshop would certainly enhance integration of the DNA barcoding in the education community.
